# Barriers and recommendations for parent–child conversations about pornography

**DOI:** 10.3389/fsoc.2024.1349549

**Published:** 2024-04-25

**Authors:** Kate Dawson, Saoirse Nic Gabhainn, Ross Friday, Pádraig MacNeela

**Affiliations:** ^1^School of Psychology, University of Galway, Galway, Ireland; ^2^Discipline of Health Promotion, University of Galway, Galway, Ireland; ^3^Psychology and Counselling, University of Greenwich, London, United Kingdom

**Keywords:** pornography, parent—adolescent relationship, adolescent—parent communication, sexual health, porn literacy, sex education, sexual information

## Abstract

**Introduction:**

Parents consistently report being worried about the impact of online pornography on their adolescent and pre-adolescent children’s development. Yet, most parents do not discuss pornography as part of parent–child conversations about sexuality. The current study sought to identify the barriers to parent–child conversations about pornography.

**Methods:**

We present two studies. The first study employed one-to-one interviews to explore parents’ (*n* = 14) beliefs about their role in their child’s pornography education. The second study involved the quantitative assessment of Study 1 findings in a sample of parents of pre-adolescent and adolescent children (*n* = 408).

**Results:**

Findings indicate that three overarching themes prevent parents from addressing pornography with their adolescent children, parents’ practical ability to discuss pornography, their attitudes toward discussing pornography, and the perceived positive impact of addressing pornography with their adolescent children. Practical ability was most often reported as the greatest barrier to parents engaging in parent–child conversations about pornography. Most notably, parents reported hesitancy in discussing pornography because they did not know how to define pornography or how to address pornography in an age-appropriate way. Fathers were also significantly less likely to believe that talking about pornography was socially acceptable.

**Discussion:**

We discuss the implications of these findings and present recommendations for developing a parents’ pornography education resource.

## Introduction

Pornography use is commonplace among adolescents in the Global North (Peter and Valkenburg, 2016). There is a growing concern among parents about the impact of early pornography engagement. Parents report concerns about other perceived high-risk areas related to sexuality (e.g., unintended pregnancy) and consequently report engaging in conversations to prevent such risks (Flores and Barroso, 2017). However, parents fail to discuss pornography, while simultaneously considering it to be a significant risk to their child (Rothman et al., 2017). Studies suggest that embarrassment and a perceived lack of urgency act as barriers to parent–child conversations about pornography. However, beyond a small number of qualitative studies, no studies have explored the barriers to parent–child conversations among a large sample of parents. The current study addresses this gap in the literature.

### Literature review

Adolescent engagement with online pornography is the norm (Peter and Valkenburg, 2016). In Western countries, the age at which individuals see pornography for the first time is decreasing, and the frequency of pornography use is increasing. Studies with Western samples of adolescents consistently show that individuals typically see pornography for the first time in early adolescence. For instance, recent studies show that approximately 80% of American high school students 70% of Dutch adolescents, 48% of 11–16-year-olds in the UK, and over 90% of Irish young adults reported that they first watched pornography (Vandenbosch and Peter, 2016; Dawson et al., 2019a; Astle et al., 2020; Martellozzo et al., 2020).

There is a growing concern among parents, educators, scholars, and policy makers about the potential outcomes associated with regularly engaging with pornography during adolescence. Although there are contradicting findings related to the outcomes of pornography use within the research literature, public opinion regarding pornography has largely remained consistent—with a large majority believing that pornography presents serious harm to the children who view it (Internetmatters.org, 2019; West, 2019). Most notably, parents worry that pornography use will contribute to a distorted view of sex and relationships, desensitize children to violence and negatively affect their understanding of consent, impact their body image and self-esteem, and lead to pornography addiction (Internetmatters.org, 2019).

Evidence suggests that parent–child conversations about pornography can have, what some authors describe as, a protective effect regarding adolescent pornography use, leading to lower rates of pornography use as well as a reduced impact of pornography on adolescent development (Wolak et al., 2007; Rasmussen et al., 2015; Rothman et al., 2017; Zurcher, 2017; Zurcher, 2019).

Parents can influence their child’s pornography use through restrictive and active mediation strategies. Restrictive mediation, including the restriction of media use or parental rules about media use is associated with less sexual media exposure (Chakroff and Nathanson, 2008). Restrictive mediation of pornography using parental blocks is relatively uncommon, with 39% of UK parents of children aged 4–16 setting controls across their broadband or mobile network (Internetmatters.org, 2019). This approach has been shown to be ineffective at preventing adolescent pornography use overall, as many youth watch pornography with their peers (Nathanson, 2001; Ševčíková and Daneback, 2014).

Active parental mediation, involving parent–child discussions impact children’s reactions to media (Chakroff and Nathanson, 2008). Active mediation has also been shown to impact the extent to which children identify with media (Rasmussen et al., 2015). Norms can be communicated through parent–child discussions or through observing parent behavior (Perkins, 2002). As such, there is some evidence that shows parents can influence their child’s attitudes toward pornography. Among a sample of American parents and their children, Rasmussen et al. (2016) found parental mediation and communication about their disapproval of pornography with their adolescent child decreased pornography use during emerging adulthood.

Parents consider discussions about early sexual debut and unprotected sexual intercourse to be important in terms of reducing perceived risks associated with adolescent sexual behavior. Consequently, parents discuss risks associated with sex significantly more than sex-positive topics (Flores and Barroso, 2017; Evans et al., 2020). Parents motivations for discussing sexual risks are often attributed to conserving the innocence of the child and ensuring that they do not engage in behavior that they are not developmentally prepared for. Parents feel more informed about some sexual topics than others. For instance, many parents feel compelled to provide their children with accurate information about sexual biology (LaSala, 2015), yet report that they have inadequate knowledge or skills when it comes to talking about pornography (Rothman et al., 2017).

There is a considerable dearth of research that sheds light on why parents avoid discussing pornography. There are several reasons why parents may not feel confident discussing pornography. First, widespread online pornography use is modern phenomenon, which parents may not have had access to in their youth. Second, pornography is often used for masturbation, a topic that many find taboo or embarrassing to discuss (Lehr et al., 2005; Noone and Young, 2010; Flores and Barroso, 2017). Some studies have identified embarrassment and a lack of relevance to young peoples lives as barriers to addressing sexual practices and sexual desire. Because pornography use relates to and involves the acknowledgement of sexual interest and desire—as pornography is typically used for arousal, this likely presents a challenge for parents who wish to discuss the perceived risks without addressing the context of pornography engagement. Parents also avoid conversations about sexuality more broadly due to fear that prompting conversations about sex, will inadvertently encourage a child’s curiosity about the topic. Finally, because pornography is typically used in private, and is often associated with shame and secrecy, parents may not be aware of their child’s pornography exposure or engagement, or indeed want to acknowledge that children can access explicit content, and therefore may not feel as though conversations at an early age are appropriate or indeed necessary for certain children. For instance, parents may be less inclined to address pornography with their daughters if they believe that pornography use is typically viewed by boys and men.

Parents may therefore need additional support to overcome learned anxiety or gendered beliefs related to discussing topics like pornography. This study seeks to explore parents’ perceived barriers to talking to their children about pornography.

## Methods

### Study design

To the best of the authors’ knowledge, no study to date has engaged parents to explore their perspectives on teenage pornography education. A qualitative approach facilitates the exploration of different parenting experiences and recommendations, allowing for a nuanced understanding of the types of relationships and contexts within the home that facilitate and block communication. Therefore, a qualitative research design was employed using the six-steps, identified by Braun and Clarke (2006) for thematic analysis.

### Inclusion criteria

Most boys and girls living in Ireland see pornography for the first time during late childhood and early adolescence (Dawson et al., 2019b). Therefore, parents of pre-teens and teenagers (ages 10–17) were invited to participate in the research.

### Participants

A total of fourteen parents (10 females and 4 males), with an age range of 31 to 58 years, participated in the one-to-one interviews. Collectively, participants were parents to 30 children, 9 were pre-teens (age 10–12) and 15 were teenagers (ages 13–17). Most participants were Irish (*n* = 11) three were non-Irish. The majority were also single or had never married. Pseudonyms are used for all participants.

### Recruitment

Recruitment posters were displayed in local community centers and on the university campus. The mature students’ society at an Irish university was also invited to share the study invitation email with their members. Potential participants were invited to email the researcher to express their interest in participating. In total 19 parents expressed interest in the study. Fourteen participants were selected by the researcher to participate in the study to ensure that the sample was diverse regarding parents’ gender and the gender and age of their teenage children.

### Materials

The one-to-one interviews were semi-structured to enable exploration of different experiences and approaches for communicating with their children. The interview schedule below was used as a guide to ensure that all research questions were touched upon. The schedule below was piloted with one female and one male parent before the study.

Research questions:

Q1. What are parents’ primary barriers to having conversations about pornography?

Q2. Do fathers perceive greater barriers to parent–child conversations about pornography?

### Procedure

All participants took part in individual one-to-one interviews. Interviews were carried out by the first author, who is experienced in working and delivering workshops and seminars to parents around adolescent sexual health. Once parents expressed interest in participating they were sent a copy of the study information sheet which provided a brief overview of the types of questions that would be asked, and important information about anonymity and confidentiality, as well as voluntary participation. Parents were provided with tea and coffee during the interview. This study was approved by the Research Ethics Committee at the university.

### Analysis

The transcripts of 14 interviews were analyzed using thematic analysis, following Braun and Clarke (2006) methodology. Data driven codes were then identified and recorded using N Vivo software (Castleberry, 2014).

## Results

### What are the barriers to parent–child discussions about pornography?

Three overarching barriers to conversations were identified: (1) practical ability, (2) attitudes toward discussion, and (3) perceived impact.

### Practical ability

Practical barriers to discussing pornography manifested in two ways. Parents refrained from beginning discussions because of difficulty in defining pornography in an age-appropriate way. Other parents prompted discussion however were met with resistance that immediately closed conversations. Practical ability was most often used to justify inaction in terms of prompting discussions about pornography.

#### Defining pornography

All parents reported that having a lack of information about how to define pornography prevented them from discussing pornography with their children. Specifically, for many parents it was the explicit nature of pornography that was difficult to explain to a child. For instance, two fathers struggled with trying to explain what they perceived to be the particularly extreme sexual nature of some pornographic content.

“*Some of the content would be too explicit for adults, let alone children, now how do I explain the concept behind this like*.” (Don)

“*You see porn could be two people making love or 10 guys in the room fucking the brains out of one woman, do you know what I mean … you are generalizing a lot*.” (Shane)

Others struggled to define pornography without presenting the topic of sex more generally, as “dirty” or unhealthy. One parent, in addressing conversations with her 13-year-old son, said that she did not know how to explain what pornography was without conflating it with sex more generally.

*“Do I say, this is like this is images of people having sex, or videos, but I don’t want to him an impression that it is completely wrong because if that is something he chooses to do when he is old enough, I do not want to have it in the back of his head, being like its seedy, but then it is a little bit seedy. So, what is porn, like do you know.”* (Pam)

#### Resistance

Parents experienced many barriers when it came to talking openly with their children about porn. Fathers to adolescent girls believed that, even though they would be interested in having discussions with their girls, believed it was the mother’s role to talk with their daughter about issues related to sexual health “*I would like to talk to her about it but I’m kind of just left a little outside the loop*,” (Don). Others were met with resistance when they attempted to talk to their child “*most times she just would not want to know*” (Joan). Some avoided discussions entirely because of the anticipated discomfort and embarrassment discussing pornography with their child would bring about “*he is certainly too embarrassed to ask me questions*” (Pam, when referring to son age 13).

### Attitudes toward discussing pornography

We identified two attitudes that acted as barriers to discussing pornography; parents reporting a lack of urgency due to the age or stage of their child, and the perception that having conversations about pornography was inappropriate.

#### Lack of urgency

There was consensus among parents that young people need to be educated about pornography, however, the information they believed was necessary to tell young people varied from parent to parent. Some parents of adolescent children believed that it was unlikely that their child had seen pornography, and therefore believed it was unnecessary to begin discussing pornography. Others believed that if they prompted conversations about pornography before the “right time” it would inadvertently direct a child to searching for pornography online. Some parents were more likely to believe their children had seen pornography. For instance, the fathers interviewed reported that it was unlikely that their daughters had seen pornography, and believed that it was best to wait until the child “needed” to have the conversation “*They do not need to know that at the start* … *I think that the limit should be, you know keeping with the curiosity of the child if they come up to you with questions*” (Don).

#### Perceived as inappropriate

Fathers were also less likely to believe that it was the role of the parent to talk to their child about pornography. Shane considered how the varied nature of pornography rendered conversations about pornography content inappropriate “*I do not want to be talking to my daughter about pornography and people having orgies and all this kind of stuff or one woman and five men in the room and that kind of crap. There is a line and for me*” (Shane).

### Perceived impact

Most parents felt there was value in children being educated about pornography “*If the parent will not talk about it then the teenager should have the option of making phone calls, anonymous but answers questions*” (Gillian). However, parents also felt adolescent pornography use was unavoidable and worried that children would not accept the information parents provided to them.

#### Adolescents are bound to see pornography

Several parents believed that there was little parents could do in terms of preventing their children from seeing pornography because of its widespread access “*We have blocks* [parental controls] *on the home computer but they are bound to see it at some stage or another*” (Pam).

#### Parents as “useless” sources of information

Parents of older adolescents believed that due to their child’s age and life stage, would not listen to their parent’s advice about pornography use. Mothers, in particular, were less likely to believe that their child would be willing to listen to parents regarding sex because they perceived them as being unable to understand the sexual lives of young people. “*What do you know you are so old you are nearly dead [laughs]*” (Joan).

### Hypothesizes

The findings from Study 1 present several questions for further investigation. We present the following hypothesize:

*H*1: Practical ability is the greatest barrier to parent–child conversations about pornography.

*H*2: Fathers will experience greater perceived barriers to conversations than mothers.

### Study 2

Study 2 involved the development of survey measures to explore the extent of parental agreement regarding the barriers and recommendations identified in Study 1 among a larger sample of parents living in Ireland. Study 2 involved two phases. The first phase involved measure development and amendment by participants in study 1 (*n* = 4) to identify a reliable set of survey items for the purpose of analysis (see measure development). The second phase involved analyzing results using the new measure as well as several additional descriptive items identified in Study 1.

## Methodology

### Participants

A total of 510 parents provided responses to the survey. However, approximately 20% (*n* = 98) did not answer survey questions beyond providing their demographic information. In addition, three participants reported being under 21 years of age and were removed from the current analysis. Most of the sample (73%) identified as female (*n* = 300; mothers), and 26% identified as male (*n* = 107; fathers). A total of 0.4% identified as non-binary, gender questioning or “other gender” (*n* = 2). The latter gender identity group was too small to provide any meaningful analysis and therefore was excluded from analysis.

The final sample consisted of 409 parents of teenagers. Participant’s ages ranged from 29 to 69. Most participants were aged in their 40s (50%), 37% were in their 50s, 4% were in their 30s and 2% were in their 60s. All participants identified as Irish. Due to the small number of non-white participants, data relating to participants is omitted. Table 1 presents information on additional demographic variables.

**Table 1:** Demographic characteristics by gender.

	Female	Male	Total
Ethnicity			
Irish	299 (90)	105 (87)	404 (89)
Marital status			
Cohabiting	11 (4)	1 (1)	12 (3)
Divorced	13 (4)	1 (1)	14 (3.5)
Married	232 (77)	87 (83)	319 (79)
Separated	16 (5)	7 (7)	23 (6)
Single	20 (7)	5 (5)	25 (6)
Widow/Widower	6 (2)	1 (1)	7 (2)
Area of residence			
Urban	201 (68)	75 (71)	276 (69)
Rural	95 (32)	30 (29)	125 (31)
Number of teenagers			
1	170 (57)	64 (61)	234 (58)
2	106 (35)	35 (33)	141 (35)
3 or more	24 (8)	6 (6)	30 (7)
Total	300	105	405

### Measure development

Items presented here were developed to reflect the primary themes and subthemes identified in Study 1. The items were reviewed by a small group of Study 1 participants for clarity and relevance.

### Barriers

#### Practical ability

A total of five items were developed to illustrate the theme “Practical Ability.” Items reflected the subthemes “Defining pornography” and “Resistance,” for example “I know how to discuss pornography with my teenager in an age-appropriate way” (Defining pornography) and “In general, teenagers want their parents to talk to them about pornography” (Resistance).

#### Parents’ attitudes toward discussing pornography

A total of five items were developed to illustrate the theme “Attitudes toward discussing pornography.” Items related to the subthemes “Lack of urgency” and “Perceived as inappropriate,” for example “Parents should discuss the topic of pornography with their teenager (s) as part of conversations about sexual health,” and “I believe that talking to my teenager about pornography is inappropriate.”

#### Perceived impact

A total of three items were developed to reflect the theme “Perceived impact.” For example, “Parents can influence their teenagers’ attitudes toward pornography.”

### Recommendations

Five items were developed, each to reflect a core recommendation presented in Study 1 for parent–child conversations about pornography.

### Procedure

#### Recruitment

Parents of teenagers aged between 13 and 18 years old were recruited through targeted advertisements on Twitter, through contacting various parents’ organizations via email and through posting study invitation links on Facebook community parenting pages. There were two rounds of recruitment. Male parents were greatly underrepresented in the first round of recruitment. Therefore, a second round, which employed the use of male-targeted ads, was conducted. All recruitment messages contained information about the aim of the study, the inclusion criteria for parent participation, a brief study overview and nature of the questions, contact information for the research team, and a link to the online survey. A detailed study information sheet was embedded in the first page of the survey. This included the aims of the study, an overview of the study questions, information regarding confidentiality and anonymity, and the risks and advantages of participation. Information on free counseling services and supports was provided to all study participants. Participants indicated their informed consent to participation by ticking a box.

#### Administration

The questionnaire was administered online and hosted on Qualtrics. Data were collected in June 2021. The questionnaire was piloted with 5 parents of teenagers to identify ambiguities or difficult questions and to ensure question and instruction clarity. Completion time of approximately 10 min was recorded and was used as an approximate time completion guideline for subsequent participants. Parents who chose to provide qualitative responses in an open text box provided took on average 3 min more to complete the survey.

#### Ethical approval

The study received approval from the Research Ethics Committee of the university and from the School of Psychology internal research ethics committee.

### Analysis

#### Missing data

There were no cases with missing values greater than 4.1%. Missing values below 5% are inconsequential to data imputation and subsequent analysis (Tabachnick and Fidell, 2007). Results from Little’s MCAR test indicated that the data were not missing at random. The missing data were imputed using the Full Information Maximum Likelihood (FIML) method in AMOS.

### Analysis

We conducted exploratory factors analysis and confirmatory factor analysis to identify a reliable set of items based on the three barrier subthemes identified in Study 1.

#### Exploratory factor analysis

We randomized and split the datasets to produce an exploratory factor analysis (EFA) and confirmatory factor analysis (CFA) dataset. We conducted EFAs using a direct oblimin rotation and maximum likelihood as a method of extraction on the EFA datasets to explore item groupings and to produce a set of items to retain in the final measures. Decisions regarding factor retention were made based on best practice recommendations (Worthington and Whittaker, 2006; Osborne and Costello, 2019). We assigned a label to each factor that reflected what each factor represented. We report measure reliability using Cronbach’s alpha coefficients, Kaiser–Meyer–Olkin test of sampling adequacy (KMO) scores (Osborne and Costello, 2019). We used Maximum likelihood (ML) as the method for data extraction. Correlations between factors were assumed, and therefore an oblique rotation with Kaiser normalization was used (Byrne, 2010). Decisions regarding factor retention were based off recommendations by Howard (2016).

#### Confirmatory factor analysis

We conducted CFA in AMOS 28 (IBM SPSS Version 28). Latent variables were created to represent the four factors identified in EFA. Maximum likelihood as a method of extraction. Following recommendations for CFA in AMOS (Byrne, 2010), CFI, TLI above IFI values above 0.95 and RMSEA below 0.06 were recommended to represent a good model fit. Item cut-off points were set at 0.32 (Byrne, 2010). Items that loaded onto latent variables below this value were removed using an iterative process. Latent variables with less than three items were omitted. The results after model modifications are presented below.

## Results

In line with the criteria for item retention outlined above, two items (Factor 2) were removed for the final EFA analysis. The KMO result from the EFA dataset was 0.734, suggesting factor analysis was appropriate for use on our data set. Anti-image matrices showed that partial correlations between variables ranged between 0.602 and 0.817. EFA produced four factors that explained 47.11% of the variance. Factor 1 contains four items; Factor 2 contained two items; Factor 3 contained three items. See Table 2 for factor scores.

**Table 2:** EFA factor loadings (F), mean and standard deviations (SD) for total measure.

Subtheme	**F1**	F2	F3	F4	M	SD
I have the words I need to discuss pornography with my teenager	**0.865**	−0.165	0.068	−0.199	3.12	1.37
I have enough information to discuss pornography with my teenager	**0.856**	−0.148	−0.013	−0.201	2.84	1.35
I know how to discuss pornography with my teenager in an age-appropriate way	**0.807**	−0.064	0.197	−0.201	3.12	1.33
I know where to get information about pornography to educate and inform my teenager	**0.746**	−0.129	0.097	−0.191	2.65	1.41
If I found out my teenager watched pornography I would punish them*	−0.143	**0.891**	0.112	0.256	1.69	0.92
If I found out my teenager watched pornography I would be angry/upset with them*	−0.136	**0.658**	0.261	0.145	2.43	1.30
Parents can delay the age that their teenager will first see pornography	−0.012	0.192	**0.747**	0.024	2.80	1.30
Parents can reduce the likelihood that their teenager will see pornography	0.001	0.278	**0.742**	0.157	3.09	1.30
Parents can influence their teenagers’ attitudes toward pornography*	0.246	−0.021	**0.458**	−0.215	4.26	0.93
I would find it strange if a parent I knew spoke to their own teenager about pornography	−0.169	0.371	−0.021	**0.705**	1.46	0.84
If I found out my teenager had watched pornography I would have an open conversation with them about it	−0.222	0.097	−0.234	**0.558**	1.58	0.82
I believe that talking to my teenager about pornography is inappropriate	−0.125	0.250	0.002	**0.555**	1.79	1.12
Parents should discuss the topic of pornography with their teenager(s) as part of conversations about sexual health.*	−0.107	−0.017	0.101	**0.444**	1.12	0.47

Items with Asterix* were removed from the final model. Items in bold correspond to the factor listed above.

### Confirmatory factor analysis

To test the proposed four-factor structure we then conducted CFA. The initial model with three latent variables resulted in a poor model fit (*n* = 280), χ^2^(41) = 87.570, *p* = 0.000, CFI = 0.929, TLI = 0.885, IFI = 0.931, RMSEA = 0.064.

We revised the model in accordance with the best practices for CFA outlined by Byrne (2010). The final model had an adequate model fit (*n* = 280), χ^2^(24) = 41.721, *p* = 0.014, CFI = 0.970, TLI = 0.944, IFI = 0.971, RMSEA = 0.051.

### Reliability

The final measure used in our analysis consisted of three factors. Factor 1 contained two items (*a* = 0.78), factor 2 contained four items (*a* = 0.89), and factor 3 contained three items (*a* = 0.54). Table 3 presents the mean and standard deviation scores for the three factors across the total sample.

**Table 3:** Unstandardized mean and standard deviation scores.

	Minimum	Maximum	Mean	Std. D
Perceived Impact	2.00	10.00	6.0201	2.23146
Practical ability	4.00	20.00	12.2011	4.60132
Social norms	3.00	14.00	4.8763	2.20343

*T*-tests. Fathers were less likely (*M* = 5.72, *SD* = 2.35) than mothers (*M* = 4.49, *SD* = 1.98) to believe that parent–child conversations about pornography were socially acceptable *t*(189) = −3.63, *p* = <0.001.

There were no significant differences reported between mothers (*M* = 12.14, *SD* = 4.68), and fathers (*M* = 12.11, *SD* = 4.30) in their beliefs about their practical ability to discuss pornography *t*(184) = 0.04, *p* = 0.972. There were no significant differences reported between mothers (*M* = 6.06, *SD* = 2.23), and fathers (*M* = 5.77, *SD* = 2.21) regarding the perceived impact of having conversations about pornography *t*(194) = 0.84, *p* = 0.403.

## Discussion

Previous studies have demonstrated that parents experience difficulties discussing pornography with their children (Rothman et al., 2017; Zurcher, 2017; Internetmatters.org, 2019). We identified three overarching barriers to discussion: parents’ practical ability, beliefs about the impact of parent–child conversations, and parents’ attitudes toward the appropriateness of discussing pornography.

Findings indicate that having a lack of practical ability about how to address pornography acts as the primary barrier to parent–child discussions about pornography. Lack of practical ability was underpinned by a lack of information about how to address pornography in an age-appropriate way. Although “embarrassment” did not feature explicitly in the interviews, how parents discussed the inappropriate sexual nature of the topic implied that, at least for some parents, embarrassment was a factor in whether they addressed pornography with their children. Previous studies identified that parents struggle to address topics related to taboo topics, like sexual pleasure and behavior as part of parent–child conversations about sexuality (Flores and Barroso, 2017; Evans et al., 2020).

The issue of pornography use is not discussed in isolation—its use relates to topics like sexual interest, pleasure, and behavior, and therefore may prove a difficult topic of conversation as a result. Lack of open parent–child communication in a home also serves as a barrier to conversations about sexuality. If parents have not established open communication with their children, beginning conversations about taboo topics will prove even more difficult. Although parents in the current qualitative study did not describe a lack of general communication as a barrier, some fathers implied that their desire to have conversations was stifled due to particular family dynamics in the home. These findings could explain why fathers in particular, reported that their beliefs about the appropriateness of conversations, acted as a greater barrier to conversations than mothers.

Parents were generally confident that parent–child conversations about pornography would be impactful. Specifically, most parents felt that conversations were needed and could protect children from perceived pornography-related harm (Rasmussen et al., 2016). However, parents also discussed the reality that there would likely be limits to parent–child discussions about pornography. Specifically, parents did not think it was appropriate to discuss the sexual acts depicted in pornography, as part of conversations.

Perceived social norms were the final barrier reported by parents. Fathers were more likely to report that social norms acted as a greater barrier to discussions, in comparison to mothers. These findings support previous research that shows fathers typically discuss issues related to sexuality less often with their children. The findings overall indicate that fathers believe in their own ability to discuss pornography, and believe in the positive impact that conversations can have, yet, may avoid conversations due to the perceived social implications of prompting conversations about pornography. In addition, Previous research has shown that parents begin talking about sex with their children when they believe that they are at an age where they start becoming sexually active. In the context of pornography, parents may be unaware that most young people first engage with pornography in childhood and early adolescence (Dawson et al., 2019a). This could prevent parents from talking about pornography as they might believe that it is an unnecessary topic to discuss with younger age groups. Raising awareness of the ubiquity of child and teenage pornography engagement may encourage parents to begin these discussions earlier.

Addressing pornography use may also have positive outcomes for adolescents (Dawson et al., 2018, 2019b; Healy-Cullen et al., 2022, 2023). Parents who address pornography can acknowledge that sexual interest and pleasure are natural parts of adolescent development and consequently help to reduce the stigma associated with sex and sexuality (Sladden et al., 2021). Indeed, studies show that reduced sexual stigma is associated with a range of positive physical and psychological health outcomes, including greater sexual decision-making abilities and safer sex practices and relationship satisfaction (Radcliffe et al., 2010; Frost and LeBlanc, 2023). However, research to date indicates that many parents discuss pornography use in a manner that may further stigmatize sex. Intervention efforts should therefore highlight the importance of avoiding moral judgments and personal discomfort when addressing pornography with their adolescent children.

### Limitations

A limitation of this study is that we relied only on parents’ retrospective accounts of their experiences conversing with their children. Parents and children can have differing opinions about the quantity and quality of parent–child conversations about sex (Flores and Barroso, 2017). It was beyond the scope of the study to involve the children of these parents in the interview process. We also discussed internet pornography in general terms, parents’ understanding and experiences with engagement with porn may vary greatly. This could account for different beliefs about the problematic qualities of youth pornography engagement. Future studies should explore parents’ understanding of pornography, and perspectives about the impact of more inclusive, less violent and ethically produced pornography. However, there was also value in talking about pornography more generally, as even though parents reported different child experiences of viewing pornography, many held the same concerns. Parents’ response to their child’s engagement may not depend on the content that they see. For example, one father saw his daughter had searched for content containing inter-family sexual relations, another parent was concerned by her child watching Japanese Hentai pornography. Both parents conveyed the same messages about pornography to their children. This could indicate that parents only feel a certain level of comfort in discussing pornography and that they may not feel equipped or desire to talk about pornography in greater detail.

### Recommendations for future research

It is recommended that interventions that aim to support parent–child communications about pornography cater to individual differences and needs. Interventions which provide a variety of methods to approach conversations, using a number of techniques, including information for short and lengthy conversations, be available in both hard and softcopy format and provide information on how to talk to different age groups will have greater reach and utility. It is also important to take the parents’ context and personal experiences of sex education into account. This may be particularly important for parents who grew up in sexually conservative households and who had little to no discussions about sex in their childhood or even throughout their adults’ lives. These parents are likely to require additional support to overcome their own discomforts around discussion sex before approaching conversations on potentially more uncomfortable or embarrassing topics, like pornography.

## Conclusion

Parents face several barriers when it comes to addressing pornography with their children. There is a need to support young people in navigating pornography and developing realistic and healthy expectations for sexual relationships (Dawson et al., 2019b). Parents are uniquely positioned to talk about sex and pornography in an individualized and private way, but they need additional support to feel prepared to discuss this often perceived taboo topic.

## Data availability statement

The datasets presented in this article are not readily available because we did not seek consent from participants to share data at the time of data collection. Requests to access the datasets should be directed to kdawson@universityofgalway.ie.

## Ethics statement

The studies involving humans were approved by the University of Galway Research Ethics Committee. The studies were conducted in accordance with the local legislation and institutional requirements. The participants provided their written informed consent to participate in this study.

## Author contributions

KD: Writing – original draft. SG: Writing – original draft. RF: Writing – review & editing. PM: Writing – original draft.
